# Plant stem and leaf segmentation and phenotypic parameter extraction using neural radiance fields and lightweight point cloud segmentation networks

**DOI:** 10.3389/fpls.2025.1491170

**Published:** 2025-03-27

**Authors:** Gaofei Qiao, Zhibin Zhang, Bin Niu, Sijia Han, Enhui Yang

**Affiliations:** Key Laboratory of Wireless Networks and Mobile Computing, School of Computer Science, Inner Mongolia University, Hohhot, China

**Keywords:** three-dimensional point cloud, plant phenotype, neural radiance fields, point cloud segmentation, lightweight network

## Abstract

High-quality 3D reconstruction and accurate 3D organ segmentation of plants are crucial prerequisites for automatically extracting phenotypic traits. In this study, we first extract a dense point cloud from implicit representations, which derives from reconstructing the maize plants in 3D by using the Nerfacto neural radiance field model. Second, we propose a lightweight point cloud segmentation network (PointSegNet) specifically for stem and leaf segmentation. This network includes a Global-Local Set Abstraction (GLSA) module to integrate local and global features and an Edge-Aware Feature Propagation (EAFP) module to enhance edge-awareness. Experimental results show that our PointSegNet achieves impressive performance compared to five other state-of-the-art deep learning networks, reaching 93.73%, 97.25%, 96.21%, and 96.73% in terms of mean Intersection over Union (mIoU), precision, recall, and F1-score, respectively. Even when dealing with tomato and soybean plants, with complex structures, our PointSegNet also achieves the best metrics. Meanwhile, based on the principal component analysis (PCA), we further optimize the method to obtain the parameters such as leaf length and leaf width by using PCA principal vectors. Finally, the maize stem thickness, stem height, leaf length, and leaf width obtained from our measurements are compared with the manual test results, yielding *R* ^2^ values of 0.99, 0.84, 0.94, and 0.87, respectively. These results indicate that our method has high accuracy and reliability for phenotypic parameter extraction. This study throughout the entire process from 3D reconstruction of maize plants to point cloud segmentation and phenotypic parameter extraction, provides a reliable and objective method for acquiring plant phenotypic parameters and will boost plant phenotypic development in smart agriculture.

## 1 Introduction

Plant phenotyping involves the systematic measurement and evaluation of various observable characteristics exhibited of plants during their growth and development. Corn, as a globally important grain crop, serves as a major food source for humans and livestock and plays a crucial role in industrial and biofuel production. In the process of breeding high-yield, disease-resistant, and high-quality corn varieties, the measurement and analysis of plant traits are essential (Wu et al., 2024). Traditional methods for extracting stem and leaf phenotypic traits primarily rely on manual operations, resulting in high labor costs, low efficiency, and potential sample damage (Tardieu et al., 2017). Additionally, manual operations have limited applicability, and cannot meet the demands of large-scale and diverse researches due to being prone to errors. In recent years, computer vision technology has made significant advances in plant phenotyping. These technologies provide automated and precise methods for data acquisition and analysis, significantly improving the efficiency and quality of plant phenotypic data collection (Rawat et al., 2022).

In previous research, 2D image-based segmentation and detection techniques have been extensively developed. Due to the relatively simple acquisition and processing of 2D images, they are suitable for large-scale data collection and high-throughput analysis and have thus been widely applied in many plant phenotyping studies (Li et al., 2020). For instance, (Zhang et al., 2024) proposed the DSBEAN framework, which combines soybean breeding technology with deep learning algorithms to predict pod counts through primary node detection and pod area identification. (Shi et al., 2022) developed a high-throughput pipeline for maize ear phenotyping, capable of extracting the number of kernels, the number of rows, and the number of kernels per row from images. (Xu et al., 2023) proposed a deep learning-based semantic segmentation model, GlandSegNet, for extracting cotton pigment gland areas. (Du et al., 2020) introduced a convolutional neural network (CNN)-based approach for object detection, semantic segmentation, and phenotypic analysis to extract the geometric and color traits of lettuce varieties. However, 2D image technology is limited by perspective constraints and the lack of depth information, leading to measurement errors, occlusion, and overlapping issues, making it difficult to accurately reflect the three-dimensional structure of plants (Qiao et al., 2023), especially the actual morphology, and characteristics of organs such as corn stems and leaves.

In 3D reconstruction, sensors are crucial for obtaining high-quality three-dimensional data. Various sensors, including monocular cameras, RGB-D cameras, LiDAR, and laser scanners, offer diverse functionalities and advantages to address different needs (Yu et al., 2024). Alongside active reconstruction methods, passive methods use RGB cameras to capture images and extract depth information, such as multiple view stereo algorithm (MVS) (Furukawa and Ponce, 2009) and structure from motion algorithm (SFM) (Moulon et al., 2013). Recent advancements in deep learning have led to the widespread application of neural networks in 3D reconstruction. MVSNet (Yao et al., 2018) enhances reconstruction accuracy by converting traditional MVS problems into deep learning tasks through an end-to-end network that generates depth maps from multi-view images. NeRF (Neural Radiance Fields) (Mildenhall et al., 2021) introduces a novel approach by using a fully connected neural network to learn implicit scene representations from multi-view images, achieving high-fidelity view synthesis and 3D reconstruction. (Thapa et al., 2018) employed a LiDAR-based sensing system to reconstruct the leaf surfaces of corn and sorghum, obtaining features such as leaf area and leaf inclination. (Hu et al., 2018) used the Kinect v2 structured light camera to measure key growth parameters of lettuce. (Wu et al., 2022) applied MVS algorithms to extract wheat point clouds and calculate parameters like leaf count, plant height, and leaf length. (Huang et al., 2024) proposed a neural distance field-based method to extract geometric parameters of walnut shells from multi-view image sequences.

Accurate phenotypic measurement requires high-quality 3D reconstruction and fast, reliable 3D segmentation methods to achieve automated extraction. Traditional point cloud segmentation methods are straightforward to implement, require minimal computational resources, allow real-time processing, and offer strong interpretability. For instance, (Miao et al., 2022) utilized TLS technology to capture point cloud data of bananas and achieved segmentation of individual banana plants using a combination of Euclidean clustering and K-means. (Wang et al., 2020) proposed a dynamic view-based adaptive K-means algorithm for segmenting and measuring the size of wheat spikes. Although these traditional methods perform well in specific scenarios, they typically involve extensive manual operation and complex parameter settings, which limits their scalability for large-scale applications. Recently, deep learning methods have been extensively applied to point cloud processing and analysis. (Yang et al., 2024b) developed a 3D semantic segmentation network for corn ears based on MVS technology to detect infected areas. (Luo et al., 2024) proposed a semantic segmentation model that combines Mask R-CNN with an improved version of PointNet++ to achieve precise segmentation of grape clusters, flower stems, and leaves. (Yan et al., 2024) developed a lightweight 3D deep learning network that accurately extracts the segmentation and phenotypic traits of corn organs.

Through these advanced methods, the potential of 3D reconstruction and point cloud segmentation technologies for agricultural phenotypic analysis has been significantly enhanced. However, practical applications still face challenges including irregular terrain, diverse crop types, and varying lighting conditions. While LiDAR devices offer high precision, they are expensive and affected by weather conditions; on the other hand, Kinect devices are cost-effective but have low point cloud resolution and are influenced by lighting conditions (Yang et al., 2024a). Compared to expensive equipment such as LiDAR, Nerfacto (Tancik et al., 2023) achieves high-quality reconstruction using only images captured by ordinary cameras, significantly reducing hardware costs. In agricultural scenarios, Nerfacto effectively addresses occlusion issues between plant leaves. Furthermore, this method requires only a small number of input images to generate high-fidelity 3D models, further enhancing data utilization efficiency. To enhance point cloud segmentation accuracy, previous research has developed complex feature extraction modules and deeper network architectures, which inevitably increase model parameters and computational demands. Due to the scarcity of high-quality plant point cloud datasets, data augmentation techniques are necessary to create more diverse training samples. To address these challenges, we first employed the 3D reconstruction method, Nerfacto, which generates high-quality plant models from a limited number of images and adapts to complex environments. Secondly, we proposed a lightweight point cloud segmentation model based on PointNet++ (Qi et al., 2017) that redesigns the encoder and decoder to significantly reduce computational complexity while maintaining accuracy. Finally, we refined the methods for calculating leaf length and width to better accommodate the curvature and complex morphology of leaves.

In summary, the contributions of this paper are as follows:

1. We applied the 3D reconstruction method Nerfacto to plant 3D reconstruction and explored methods for extracting point clouds from implicit neural radiance fields (NeRF). Leveraging Nerfacto technology, we developed a 3D point cloud dataset for maize stalks and leaves, including segmentation and related phenotypic data for 20 plants.
2. We designed a lightweight plant point cloud segmentation model with only 1.33 M parameters. We introduced a novel Global-Local Set Abstraction (GLSA) module to integrate local and global information and an Edge-Aware Feature Propagation (EAFP) module to enhance the handling of deep-edge features. Experimental results show that our proposed model while maintaining the smallest parameter count, achieves competitive accuracy compared to the latest segmentation networks across multiple datasets (maize, tomato, soybean).
3. We proposed an optimized method for better obtaining parameters such as leaf length and leaf width by segmenting the PCA principal vectors. This approach generates curves that accurately reflect leaf curvature, allowing for a more precise measurement of leaf length and width. This method provides robust support for the measurement of plant phenotypic parameters.

## 2 Materials and methods

### 2.1 Overview

**Figure 1:**
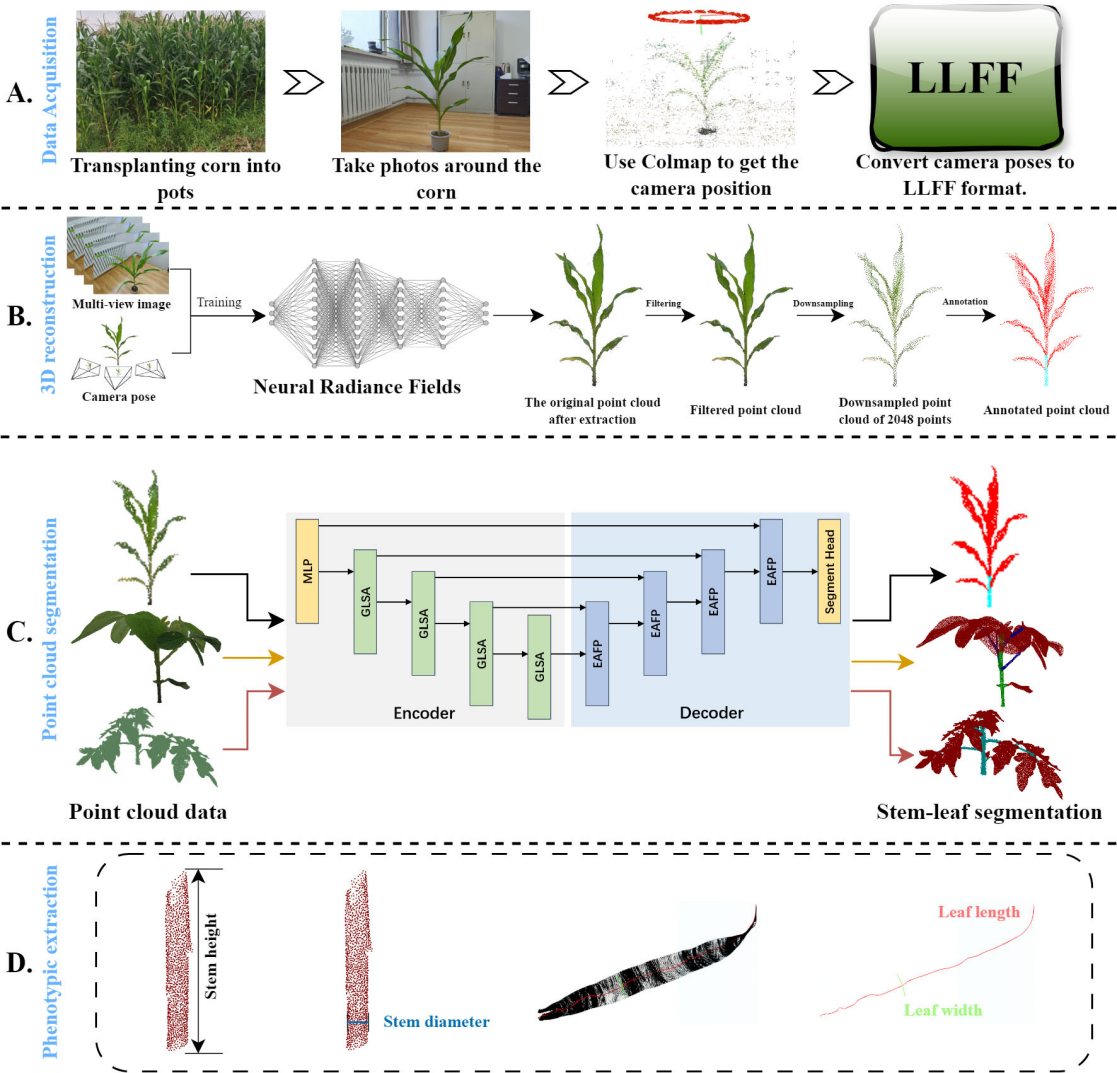
Overview of the proposed framework. **(A)** Photographs were taken around a maize plant. The bitmaps of these image frames were computed using COLMAP and then converted to the Local Light Field Fusion (LLFF) format. **(B)** The converted data trains the neural radiance field, which generates dense point clouds. These dense point clouds are then preprocessed. **(C)** The main stems and leaves of the maize are segmented using PointSegNet. The segmentation effectiveness of the model is verified using various complex plants. **(D)** Based on the segmentation results of maize plants, four phenotypic traits (stem height, stem thickness, leaf width, and leaf length) were extracted to validate further the point cloud segmentation effect and shape extraction methods.

The overall workflow is illustrated in **Figure 1** and is divided into four parts: data acquisition, a 3D reconstruction based on neural radiance fields, a lightweight plant point cloud stem and leaf segmentation model, and plant phenotypic data measurement. Firstly, in part (A), a smartphone is used as the image acquisition device, and it is handheld and slowly moved counterclockwise around the maize plant to ensure the capture of clear and complete coverage videos of the entire plant. Several image frames were extracted from the videos, and COLMAP is used to compute the poses of the camera to determine its position and orientation in space. To accelerate the convergence speed of the neural radiance fields and improve the reconstruction quality, these images and their associated camera poses and parameters are converted into Local Light Field Fusion (LLFF) format (Mildenhall et al., 2019). Secondly, in the part (B), a neural radiance field (NeRF) is trained by using the transformed data, in which NeRF can capture rich 3D structural information by computing depth estimations of the plant surface from each viewpoint. Repeating these steps generates a dense point cloud so that the point cloud of entire maize plant is obtained, and then processed using statistical filtering and farthest point sampling (FPS). Thirdly, in the part (C), the processed point cloud is fed into the proposed lightweight stem-leaf point cloud segmentation model to obtain segmentation results of them. Experiments are also conducted on the tomato and soybean datasets to further validate the proposed segmentation model performance across various plant types. Finally, key four maize phenotypic traits including the stem height, stem diameter, leaf width, and leaf length are extracted from the segmented point clouds in the part (D).

### 2.2 Experimental materials and data collection

#### 2.2.1 Maize point cloud collection and annotation

We randomly selected 20 maize plants from the experimental field at Inner Mongolia University and transplanted them indoors for data acquisition. During the acquisition process, we ensured adequate lighting and wind-free conditions to minimize the impact of lighting and wind on data quality. A Xiaomi 14 smartphone was handheld and moved counterclockwise around the plants to capture video. The capture time for each maize plant was set to 40-50 seconds, ensuring comprehensive coverage from all angles. Ultimately, 60 to 80 key image frames were extracted from the videos. Next, we used COLMAP to process these image frames and calculate the camera poses, i.e., the position and orientation of the camera in space. To support subsequent 3D reconstruction and analysis, we converted the images and their associated camera poses and parameters into the Local Light Field Fusion (LLFF) format. We then used Nerfacto for 3D reconstruction to obtain dense point cloud data (detailed in section 2.3).

To validate the model’s applicability across different datasets and further expand the dataset, we combined the collected point cloud data with a publicly available maize dataset (Yang et al., 2024c), and constructed a new dataset with 448 point cloud samples. To ensure consistency in format, data quality, and annotations between the newly collected maize point cloud data and the public maize dataset, we implemented the following measures:

1. Color Threshold Segmentation: We used color threshold segmentation to distinguish between the ground and maize plants. First, we calculated the histogram of the color values and then applied Otsu’s method to determine the optimal threshold for effectively segmenting the non-ground areas from the ground.
2. Statistical Filtering for Denoising: We used statistical filtering to remove noise by calculating the distance of each point within its neighborhood, then removed the noise points that exceeded this threshold. This method effectively eliminates isolated points and random noise while preserving the main structure.
3. FPS Downsampling: We applied the Farthest Point Sampling (FPS) algorithm to downsample the point cloud. This algorithm iteratively selects the farthest points to ensure that the downsampled point cloud is uniformly distributed while maintaining geometric features. This approach reduces data volume while retaining the overall shape and key details of the point cloud of maize plant.
4. Point Cloud Labeling: We used a point cloud labeling tool to manually label the stem and leaf organs of maize as ground truth for model training.

#### 2.2.2 Tomato and soybean point cloud dataset

Due to the relatively simple structure of maize plants, we further evaluated the performance of our proposed point cloud segmentation model on different plant species using two publicly available plant point cloud datasets with more complex structures: Pheno4D (Schunck et al., 2021) and Soybean-MVS (Sun et al., 2023). The Pheno4D dataset is a sub-millimeter precision plant point cloud dataset obtained using a laser triangulation scanner. It includes 77 point cloud samples from seven tomato plants collected over 20 days, manually labeled into three categories: soil, stem, and leaves. The Soybean-MVS dataset is a 3D point cloud dataset covering the entire growth period of soybeans, reconstructed using multi-view stereo (MVS) technology. It contains 102 samples of five soybean varieties at 13 growth stages, manually labeled into three categories: leaves, main stem, and branches, including x, y, z coordinates and r, g, b color values. To ensure that the network focuses more on the segmentation of plant organs during training, we preprocessed the point clouds of tomato and soybean plants. Specifically, we removed the “soil” points from the tomato plant dataset, and specific color attributes from the soybean plant dataset. Thus, each point cloud includes x, y, z coordinates and normal vector information *N_x_*, *N_y_*, *N_z_*, calculated through normal vector computation.

### 2.3 3D reconstruction based on Nerfacto

#### 2.3.1 Implicit neural representations

**Figure 2:**
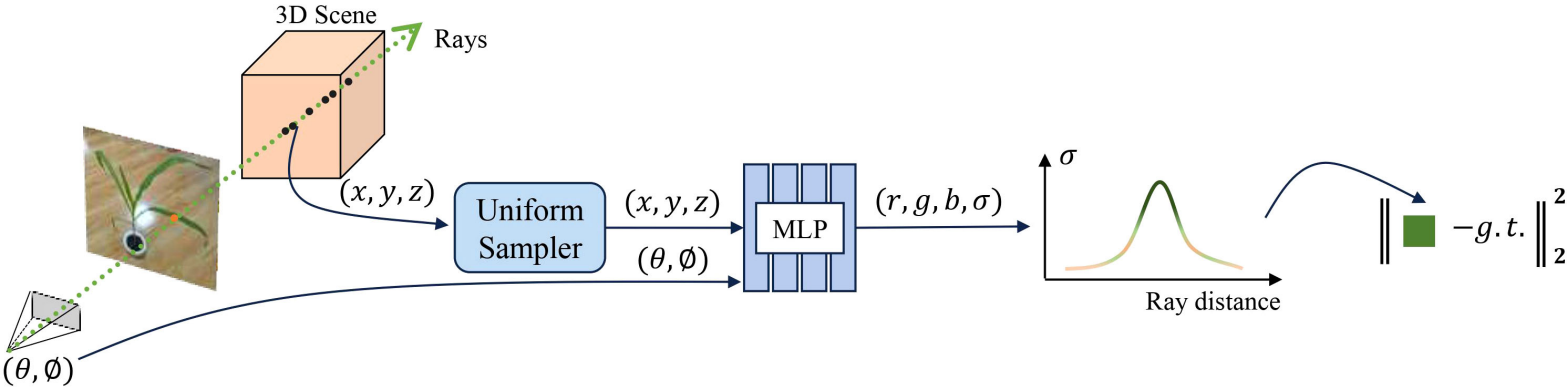
NeRF-based 3D representation pipeline.


In traditional 3D representation methods, we typically use point clouds, meshes, or voxels to explicitly define the geometric information of an object, including its vertices, edges, faces, and topological relationships. In contrast, implicit representation methods describe the geometric shape and appearance of a 3D model through a function that uses coordinates as input. This kind of approach does not require the explicit definition of geometric structures but instead predicts surface attributes such as color and density through an implicit function. Neural Radiance Fields (NeRF) (Mildenhall et al., 2021) is an application of implicit neural representation specifically designed for high-quality 3D scene reconstruction and novel view synthesis. NeRF employs a multilayer perceptron (MLP) to process the input 3D coordinates and view directions to predict the color and density of each point, as illustrated in **Figure 2**. Specifically, NeRF first casts a ray *r*(*t*) = *o* + *t*d from a given camera position *o* = (*x*_0_ *,y*_0_ *,z*_0_) along the viewing direction d. Along each ray, a series of sample points are uniformly sampled or using stratified sampling within a predefined depth range to obtain their coordinates. These coordinates, along with the view direction, are input into the MLP, which outputs the color and density for each sampled point. Finally, the volume rendering techniques integrate these color and density values along the ray to obtain the color value of the corresponding pixel in the image, as shown in Equation 1. During NeRF training, the MLP weights are optimized by minimizing the photometric loss, defined as the mean squared error between the rendered image and the actual observed image, to ensure that the generated images are as consistent as possible with the real observations.

(1) $$C(r)=\int_{t_{1}}^{t_{2}}T(t)\cdot \sigma (r(t))\cdot c(r(t),d)\cdot dt$$

The transmittance function *T*(*t*) represents the probability that a ray is not occluded by an object at a distance *t*. In physics, we assume that the scene is composed of a collection of luminous particles, with the density field *σ*(*x*) describing the probability of a ray encountering particles at position *x*. The transmittance function *T*(*t*) is described in Equation 2 as follows:


(2) $$T(t)=\text{exp }(-\int_{t_{1}}^{t}\sigma (r(u))\cdot du)$$

In the field of agricultural 3D reconstruction, NeRF can capture plant structures and scene details more accurately than traditional methods, providing more realistic and detailed reconstruction results. It is capable of adapting to complex vegetation structures and various farmland environments. However, the training and inference processes of NeRF typically require substantial computational resources and time, which do not fully align with the practical needs of agricultural production. Moreover, NeRF generates implicitly represented 3D scenes, which differ from traditional point cloud or mesh models, leading to challenges in integrating them with existing agricultural analysis tools.

To address the aforementioned issues, we chose an improved version of NeRF, the Nerfacto model, as our 3D reconstruction tool. Nerfacto retains the fine reconstruction capabilities of NeRF while optimizing computational efficiency and resource utilization, making it better suited for the practical needs of agricultural applications. Next, we will discuss the structure of the Nerfacto model in detail and how to extract point cloud data from its implicit representation to effectively apply it to plant phenotyping tasks.

#### 2.3.2 Network structure of Nerfacto and point-cloud extraction method

**Figure 3:**
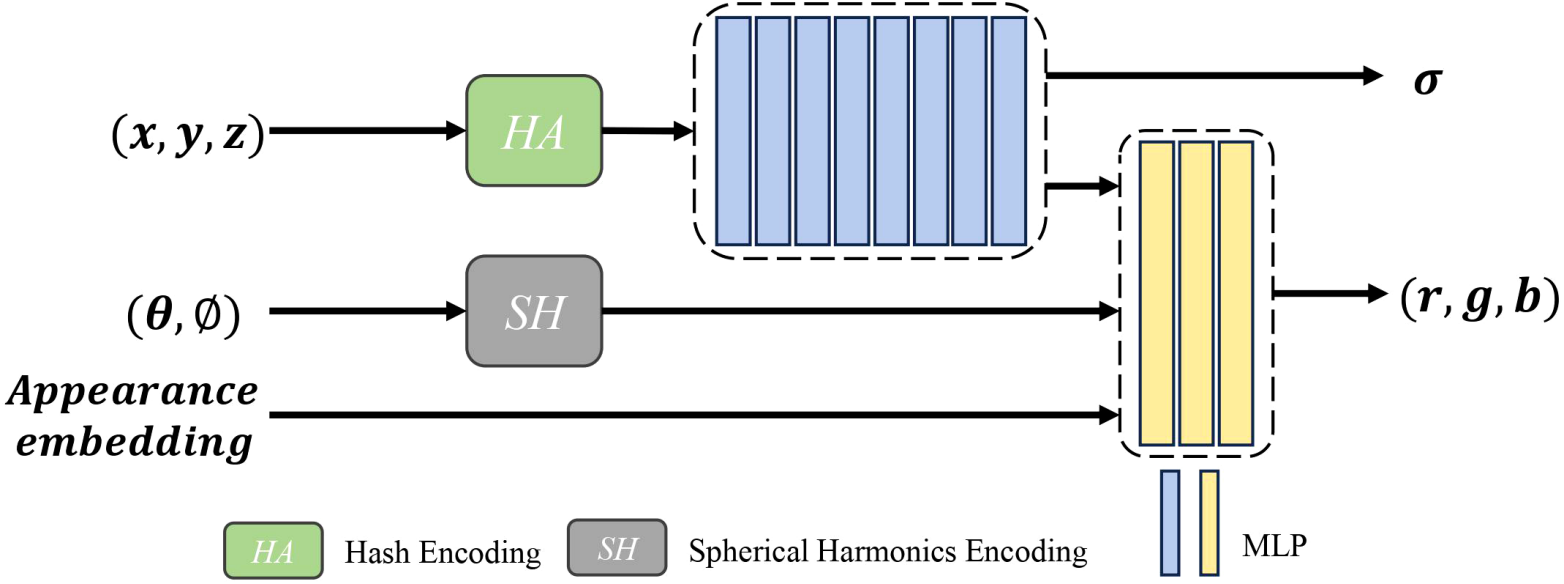
Structural components within the Nerfacto model.

To enhance the efficiency and effectiveness of the sampling process, as shown in **Figure 3**, Nerfacto (Tancik et al., 2023) employs a segmented sampling strategy. This sampler performs uniform sampling within a fixed distance from the camera and applies distributed sampling at greater distances. This approach not only maintains dense sampling for nearby objects but also improves sampling efficiency for distant objects. Additionally, the sampling process emphasizes regions of the scene that contribute most to the final rendering. Unlike NeRF, which uses an MLP model for predictions, Nerfacto processes the input 3D coordinates and view directions by combining hash encoding (HA) and spherical harmonic encoding (SH), and can also predict the scene’s density (*σ*) and color (r, g, b). Moreover, the appearance embedding vectors further enhance the model’s adaptability to different viewpoints and lighting conditions. These improvements make Nerfacto significantly superior to NeRF and other NeRF-based models in terms of accuracy, efficiency, and applicability, We thought that this makes it better suited for practical agricultural 3D reconstruction applications.

For each ray, we have a set of sample points, with each point having a known depth *d_i_*. The model, once trained, learns the density and color information of each sample point in the scene. Using the volume rendering equation, it calculates the weight *w_i_* for each sample point. Specifically, as derived from Equation 2, the accumulated transmittance *T_i_* for each sample point represents the probability that the light has not been obstructed from the start of the ray to the current point. For the *i* − *th* sample point, the accumulated transmittance is computed as shown in Equations 3, 4, where the transmittance *α_i_* of each sample point is derived from its density *σ_i_* and the sampling interval *δ_i_*. Thereby, from these calculations, the formula for the weight *w_i_* can be determined, as shown in Equation 5.


(3) $$T_{i}=\prod_{j=1}^{i-1}(1-\alpha_{i})$$

(4) $$\alpha_{i}=1-\text{exp }(-\sigma_{i}\cdot \delta_{i})$$

(5) $$w_{i}=\prod_{j=1}^{i-1}\text{exp }(-\sigma_{i}\cdot \delta_{i})\cdot (1-\text{exp }(-\sigma_{i}\cdot \delta_{i}))$$

Finally, the formula for computing the expected depth $t_{surface}$ is given as shown in Equation 6.


(6) $${\text{t}}_{surface}=\frac{\sum_{i=1}^{N}d_{i}w_{i}}{\sum_{i=1}^{N}w_{i}}$$

where N is the number of points sampled along the ray.

After obtaining the approximate depth, the next step is to compute the position of the point on the ray *r*(*t*) = *o* + *t* **d** within the world coordinate system, as shown in Equation 7.


(7) $$p=o+t_{surface}\times d$$

where *p* represents the position of the 3D point, o is the spatial coordinate of the camera, and d is the direction of the ray.

Once the 3D position is determined, the color value at that location can be directly queried from the trained model. At this stage, the generated point cloud reflects only a single viewpoint of the original scene. By repeating these steps for each pixel across multiple images, and mapping the corresponding coordinates and color information of each sample point in the scene to the world coordinate system, a dense point cloud is generated (Hu et al., 2024). This process can completes the 3D reconstruction of the plant.

### 2.4 Lightweight plant point cloud segmentation network

#### 2.4.1 Network architecture

PointNet++ (Qi et al., 2017) is a deep learning model designed for processing point cloud data and represents an advancement and refinement of PointNet. It introduces a hierarchical structure for handling point cloud data, including sampling layers, feature extraction layers, and aggregation layers. Each layer is responsible for processing point cloud information at different levels of granularity, progressively extracting higher-level features and thus enhancing the model’s ability to learn and analyze point cloud features. However, despite the multi-scale processing introduced by PointNet++, the integration of global information may be insufficient, leading to a limited understanding of the overall point cloud structure and impacting the accuracy of analysis and predictions. Especially, for point clouds with sharp edges or complex local shape variations, PointNet++ may struggle to accurately capture and analyze local features, resulting in decreased segmentation and classification precision in these areas. To achieve strong generalization and performance, PointNet++ requires a substantial amount of labeled data for training to enhance model effectiveness and stability.

**Figure 4:**
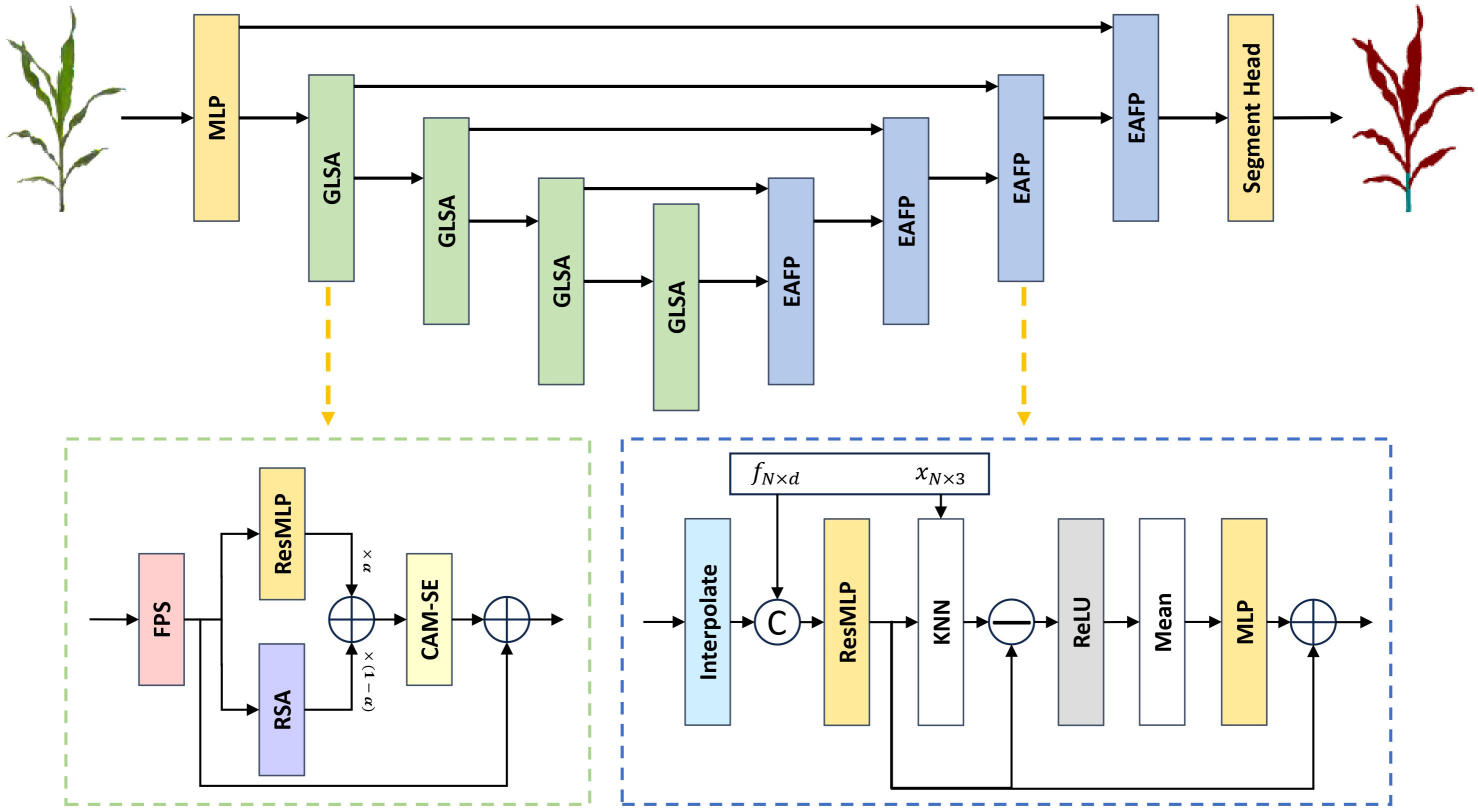
The overall architecture of PointSegNet, a U-net style architecture, has a Global-Local Set Abstraction (GLSA) module for downsampling and an Edge-Aware Feature Propagation (EAFP) module for upsampling. The *x_N_*_×3_ and *f_N_*_×_ *_d_* in the Edge-Aware Feature Propagation (EAFP) module are jump connections from the encoder. *x_N_*_×3_ denotes the spatial location information of the point cloud and *f_N_*_×_ *_d_* denotes the feature information of the point cloud.

In recent years, the field of point cloud segmentation has emerged in many advanced feature extraction modules and model architectures. However, these complex structures often result in increased model parameters and computational costs (Guo et al., 2020), in contrasts with the need for lightweight and practical solutions for agricultural applications. To address this, we propose a lightweight plant organ segmentation network called PointSegNet. As illustrated in **Figure 4**, it follows a conventional encoderdecoder framework. Initially, data is processed through a multi-layer perceptron (MLP) module for preliminary feature extraction, mapping low-dimensional features to a higher-dimensional space. In the encoder section, we have redesigned the structure and introduced the Global-Local Set Abstraction (GLSA) module, which integrates both local and global information. The encoder, consisting of four GLSA modules, enhances the network’s ability to capture point cloud features, extract multi-scale information, and reduce information loss during compression. Thereby, they improve the network’s generalization and adaptability. For the decoder section, we have restructured the design and proposed the Edge-Aware Feature Propagation (EAFP) module, which is used for edge-preserving sharpening. This module can retains effectively and recovers detail features from the original resolution, thereby improving the model’s understanding of semantic information, especially in terms of the detail and edge preservation. The network’s final layer employs a segmentation head composed of two MLP layers to predict semantic labels for each point. Additionally, inspired by PointNext (Qian et al., 2022), we incorporate some advanced training strategies, including data augmentation techniques (such as point re-sampling, jittering, random scaling, and rotation) and some optimization strategies (such as loss functions, optimizers, learning rate scheduling, and hyperparameter tuning). These improvements significantly reduce the model’s reliance on the quantity of training data and effectively mitigate overfitting issues, particularly in scenarios with limited data samples.

#### 2.4.2 GLSA module for capturing local and global structural information in point clouds

As illustrated in **Figure 4**, the proposed Global-Local Set Abstraction (GLSA) module comprises four components: the FPS downsampling layer, the ResMLP module, the Relative Spatial Attention (RSA) module, and the Channel Attention Mechanism with Statistical Enhancement (CAM-SE). Initially, the FPS downsampling layer performs subsampling of the input point cloud using the farthest point sampling at a rate of 0.5. Subsequently, the ResMLP module and the RSA module extract the local and global features from the downsampled point cloud, respectively. Finally, the CAM-SE module is employed to mitigate the impact of noise and irrelevant information.

**Figure 5:**
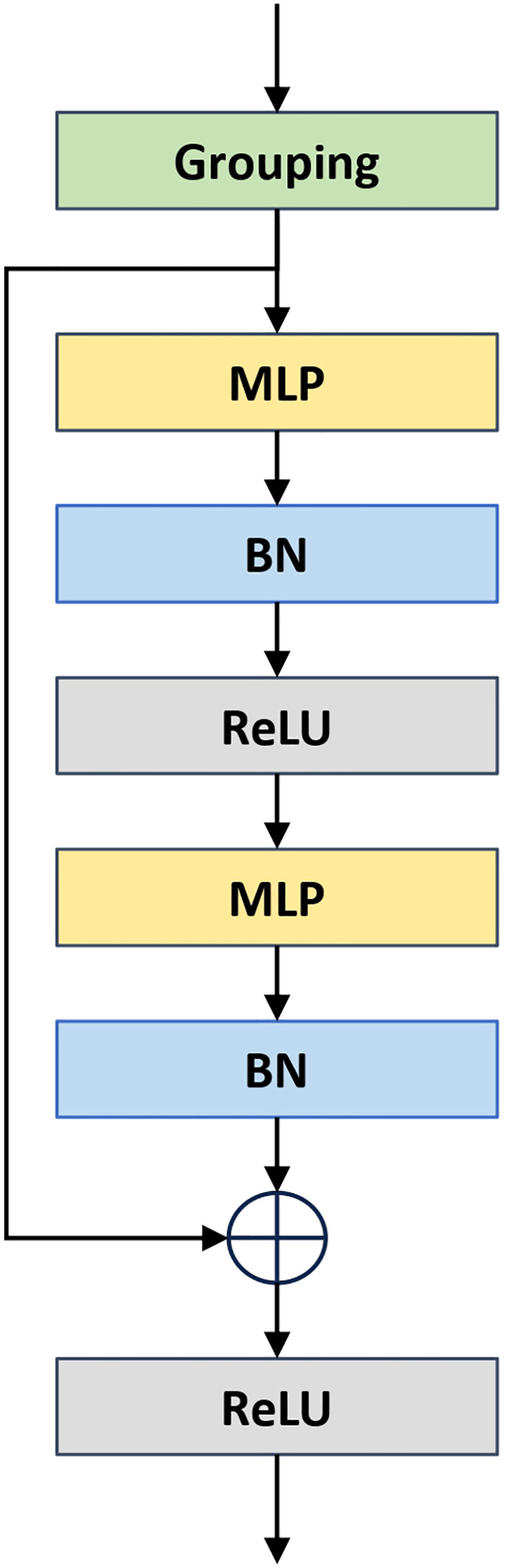
The illustration of Residual Multi-Layer (ResMLP) module.

In the ResMLP module, as illustrated in **Figure 5**, the process begins with a ball query to obtain the k nearest points for each sampled point, thereby partitioning the point cloud data into multiple local regions. Subsequently, a two-layer MLP structure, implemented using 1×1 convolutions, projects the features of each point into a higher-dimensional space along the channel dimension, which enhances the network’s ability to represent local features. Residual connections enable the model to capture deeper features and help mitigate the issue of gradient vanishing.

**Figure 6:**
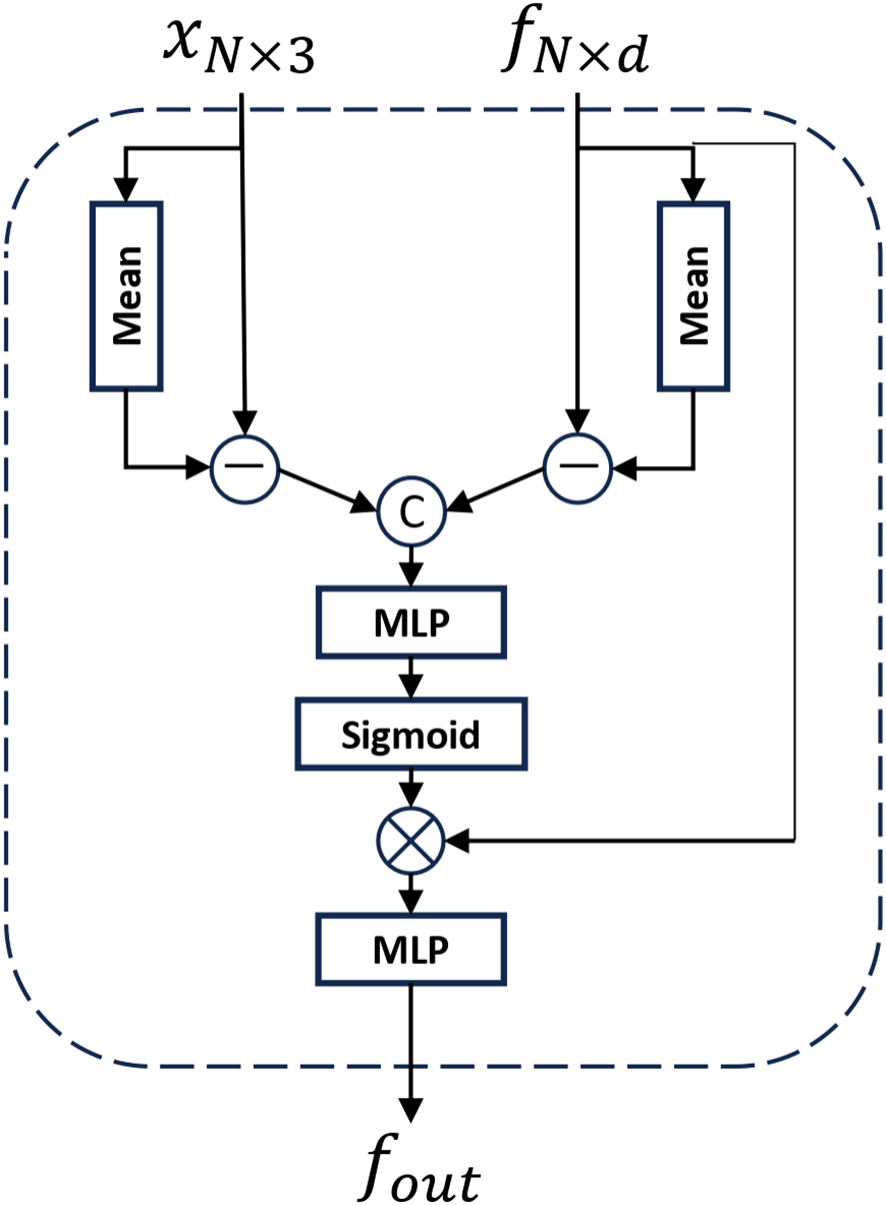
The illustration of Relative Spatial Attention (RSA) module. where *x_N_*_×3_ represents the spatial location information of the point cloud and *f_N_*_×_ *_d_* represents the feature information of the point cloud.


As shown in **Figure 6**, the Relative Spatial Attention (RSA) module differs from standard global attention mechanisms by leveraging spatial and semantic proximity, and approximates the entire shape using only the currently downsampled points, which significantly reduces computational costs. For the input point cloud data *p_in_* and *f_in_*, the coordinates of all points are first averaged to obtain a global mean point *P*, serving as the global positional encoding of the point cloud. Concurrently, the features of each point are averaged to obtain a global feature mean *F*, providing a global perspective for understanding point cloud features. Each point’s coordinates are then subtracted from the global mean *P*, yielding deviation vectors representing each point’s position in the global coordinate system. Similarly, each point’s features are subtracted from the global feature mean *F*, yielding deviation features representing local feature variations relative to the global context. These deviation vectors *P* and deviation features *F* are concatenated and input into a multi-layer perceptron (MLP). After passing through a sigmoid function, the attention weights for each point are obtained. The original point cloud features are multiplied by these attention weights and processed through an MLP layer again to yield the final output of the global attention, as shown in Equations 8 to 10. By computing the global mean point and feature mean and determining the positional and feature deviations of each point relative to the global context, this module enhances the understanding of each point’s global positional relationships and local feature variations, thereby improving the overall expressive capability of the point cloud.

(8) $$P=p_{in}-\frac{1}{N}\sum_{i=1}^{N}p_{in}^{i}$$

(9) $$F=f_{in}-\frac{1}{N}\sum_{i=1}^{N}f_{in}^{i}$$

(10) $$f_{RAS}=MLP_{2}(f_{in}\cdot Sigmoid(MLP_{1}(P,F)))$$

Finally, we use a learnable parameter *X* to fuse local and global features to control their contributions during the fusion process. This allows the model to adjust to maximize the overall effectiveness of feature representation automatically. Given that each point in the point cloud data may have varying importance and contribution, we employ a CAM-SE module to dynamically adjust the significance of each feature channel using statistical information (mean and standard deviation) along with learned parameters. Specifically, for the input point cloud features *f*, we first calculate the mean and standard deviation of each channel across the entire point cloud and concatenate them into a new tensor *t*. We then apply a learnable parameter matrix to weight the tensor *t*, sum the weighted results along the channel dimension, and process them through batch normalization followed by a Sigmoid activation function to obtain the final attention coefficients *g*. The point cloud features *F* are then multiplied by the attention coefficients *g*, and a residual connection is added to the features *F*, resulting in an enhanced feature representation through weight adjustments, as described in Equations 11–14.


(11) $$u_{i}=\frac{1}{N}\sum_{j=1}^{N}f_{ij}$$

(12) $$\sigma_{i}=\sqrt{\frac{1}{N}\sum_{j=1}^{N}{\left(f_{ij}-u_{i}\right)}^{2}+Є}$$

(13) $$g_{i}=Sigmoid(BN(\sum_{j=1}^{N}([u_{i},\sigma_{i}]\cdot W_{i})$$

(14) $$f_{\text{CAM}-\text{SE}}=g\cdot f+f$$

#### 2.4.3 EAFP module for edge-aware capability

In point cloud segmentation models such as PointNet++, the decoder module has played a crucial role in the final segmentation results. It is responsible for progressively up-sampling the multiscale features extracted by the encoder, to recover them to the same resolution as the input point cloud, and finally generating predictive labels for each point. The design and implementation of the decoder module directly impact the accuracy and quality of the segmentation results. The process can be summarized as follows: firstly, the lower-resolution features are upsampled to higher resolutions using nearestneighbor interpolation; next, the upsampled features are fused with the skip connection features from the corresponding layers of the encoder; finally, the fused features undergo nonlinear transformation and processing through multilayer perceptrons (MLPs) to map the features to the desired output space (e.g., for classification, segmentation, or generation tasks).

In traditional decoder module designs, nearest-neighbor interpolation easily results in unsmooth interpolation outcomes, and introduce high-frequency noise, so that it makes the network training process difficult to converge, and disrupts the geometric consistency of the point cloud. It is the reason that we propose the Edge-Aware Feature Propagation (EAFP) module. Previous research has shown that edge-aware methods based on EdgeConv (Wang et al., 2019) can better extract local geometric structures through convolution operations. However, when handling deeper edge features, these methods can lead to increased memory consumption during training and inference.

As shown in **Figure 4**, The structure of our proposed Edge-Aware Feature Propagation (EAFP) module is illustrated as follows: First, upper-layer features are expanded to the current resolution through interpolation and then fused with the skip connection features *f_N_*_×_ *_b_* from the corresponding encoder layer to retain high-level semantic information. Next, the features are further processed by ResMLP to enhance their expressive power and non-linear characteristics. Subsequently, by incorporating the positional information *x_N_*_×3_ of the skip connection points, K-Nearest Neighbors (KNN) is used to retrieve the *K* = 16 nearest neighbor features for each point. The original features are then subtracted from these nearest neighbor features to obtain edge features, which are smoothed and enhanced using ReLU activation and Mean operations, improving robustness to outliers (Xiu et al., 2023). Finally, a layer of MLP maps the processed features to the final output space. This module leverages the spatial relationships between points in the point cloud to enhance task-relevant edges while smoothing irrelevant outliers, thereby better preserving and processing useful information and reducing the interference of unrelated elements on the results.

#### 2.4.4 Loss function

During the model training process, we use PolyFocalLoss (Leng et al., 2022) as the loss function, as shown in Equation 15. PolyFocalLoss is an advanced loss function that integrates focal loss with a polynomial weighting mechanism. This combination effectively addresses the class imbalance in point cloud segmentation, significantly enhancing the model’s ability to learn from challenging samples and improving segmentation accuracy.


(15) $$L_{Ploy}=L_{FL}+Є\cdot {\left(1-p_{t}\right)}^{\gamma +1}$$

In this context, *L_FL_* represents the focal loss function, *P_t_* denotes the weighted sum of the predicted probabilities and the true labels, *γ* is the focus factor, which is used to adjust the weights of the difficult and easy recognition samples, and *Є* is a small constant used to prevent numerical instability or excessively small values. The specific process is detailed in Equations 16 to 18.


(16) $$L_{FL}=L_{CE}\cdot {\left(1-P_{t}\right)}^{\gamma }$$

(17) $$L_{CE}=-[labels\cdot \text{log }(p)+(1-labels)\cdot \text{log }(1-p)]$$

(18) $$P_{t}=labels\cdot p+(1-labels)\cdot (1-p)$$

Among them, *p* is the predicted probability after activation by the sigmoid activation function.

#### 2.4.5 Experiment settings and evaluation metrics

We will train and test the point cloud segmentation network on three different plant point cloud datasets. To ensure full utilization of the data, we divided the datasets into an 80% training set and a 20% validation set. Before inputting the model, we selected 2048 points using an FPS downsampling strategy to serve as the number of input data points for the model. We implemented our method on a single NVIDIA GTX 3060Ti GPU using the PyTorch framework. During training, the batch size is set to 4. We choose the AdamW optimizer and set the weight decay to 1 × 10^−4^ to optimize the parameters in training and effectively suppress overfitting. The training period of the experiment is 300 epochs. To manage the learning rate more accurately, we adopt the multistep scheduler. During the training process, the learning rate is decayed at a rate of 0.1 when 210 and 270 epochs are reached. This decay strategy of multistep learning rate helps to maintain good convergence of the model at different training stages.

For data augmentation, we use a combination of random scaling, normalization, and jittering techniques. Specifically, random scaling adjusts the scale of each point cloud to a range between 0.8 and 1.2, aiding the model in learning features at various scales. Point clouds are also centralized and normalized to ensure consistency in the coordinate system and numerical range across all data before training. To introduce additional randomness and noise, we apply jittering technique to each point by adding independent Gaussian noise, with a standard deviation of 0.001 and a noise threshold of 0.005. This can simulates real-world noise and uncertainty, enhancing the model’s robustness to noise and variations. The effectiveness of these data augmentation methods in improving point cloud segmentation accuracy has been validated in the PointNeXt (Qian et al., 2022) model.

This paper evaluates the performance of our proposed point cloud segmentation network, PointSegNet, by using a comprehensive set of metrics, including Intersection over Union (IoU), Precision, Recall, and F1-score, as well as model parameter count and computational complexity. Among them, IoU measures the overlap between the predicted region and the ground truth region, with higher values indicating greater alignment between the prediction and the actual situation. Precision assesses the proportion of true positive samples among all samples predicted by the model as positive class, reflecting the accuracy of the model’s predictions. Recall measures the proportion of actual positive samples that are correctly identified by the model, highlighting its ability to detect all positive samples. The F1 score combines Precision and Recall into a single metric, providing a balanced evaluation of the model’s performance in predicting both positive and negative samples. Specifically, the formulas for calculating these evaluation metrics are as shown in Equations 19–22.


(19) $$Precision=\frac{TP}{TP+FP}$$

(20) $$Recall=\frac{TP}{TP+FN}$$

(21) $$F1=\frac{2Precision\times Recall}{Precision+Recall}$$

(22) $$IoU=\frac{TP}{TP+FP+FN}$$

Where TP (True Positive) denotes the number of positive class samples correctly predicted by the model, FP (False Positive) denotes the number of samples where the model incorrectly predicted a negative class as a positive class, and FN (False Negative) denotes the number of samples where the model failed to predict a positive class as a negative class.

#### 2.4.6 Phenotypic trait extraction and evaluation

We extracted four phenotypic parameters—stem height, stem diameter, leaf length, and leaf width—from the segmented maize stems and leaves to evaluate the effectiveness of our proposed point cloud segmentation model (PointSegNet), in plant phenotyping tasks. The detailed parameter extraction process is as follows:

1. Stem height: Before measurement, the point cloud rotation correction is necessary. The principal direction of the stem is determined using PCA (Principal Component Analysis), and a rotation matrix aligning this direction with the Z-axis is computed. The entire point cloud is then rotated to be parallel to the Z-axis. Finally, the stem height parameter is obtained by subtracting the minimum z-value from the maximum z-value within the stem point cloud.
2. Stem diameter: First, the point cloud data is segmented along the Z-axis into four equal-height parts. The lowest segment is selected for analysis because the base of the stem is usually the most stable and uniform. Next, the linear fitting is performed on the point cloud data of the lowest z-value segment using the least squares method. Specifically, a linear regression model fits a plane that describes the variations in z-values in the X and Y directions, respectively. Then, we calculate the vertical distance from each point to the fitted plane (i.e., the difference between the actual z-value of the end and the z-value predicted in the plane). The stem diameter is defined as twice the median of these residuals’ absolute values, as the median is insensitive to outliers and can more accurately reflect the true diameter of the stem (Miao et al., 2021).
3. Leaf length and width: To extract leaf length and width from the leaf point cloud, PCA (Principal Component Analysis) is first used to determine the primary direction of the point cloud, and the leaf point cloud is projected onto this principal vector. The point cloud is then segmented based on these projection values. For each segment, the points corresponding to the minimum and maximum projection values are identified, as the extreme positions along the principal direction, i.e., the two most distant points. To accurately fit the leaf surface, multiple points are uniformly inserted between the start and end points, then projected onto the leaf point cloud. By connecting the start point, endpoint, and projection points, the width of the segment is determined. The maximum width among all segments is taken as the leaf’s width. For leaf length calculation, the midpoints of the start and end points of each segment are identified and projected onto the leaf point cloud. By connecting all projection points, the total length of the leaf is obtained. Finally, smoothing is applied to remove noise and irregularities in the leaf length and width contours to improve measurement accuracy and data continuity. Later experimental result show that our method can be responsible for the more stable and reliable calculations of leaf width and length.

To assess the accuracy of the phenotypic parameters extracted from the PointSegNet model segmentation, we compared the predictions calculated from the model segmentation results with the actual measurements by measuring the actual values and calculating the *R* ^2^ coefficients and the RMSE, as shown in Equations 23, 24.


(23) $$R^{2}=1-\frac{\sum_{l=1}^{n}{\left(v_{l}-\hat{v_{l}}\right)}^{2}}{\sum_{l=1}^{n}{\left(v_{l}-\bar{v_{l}}\right)}^{2}}$$

(24) $$RMSE=\sqrt{\frac{1}{n}\sum_{l=1}^{n}{\left(v_{l}-{\hat{v}}_{l}\right)}^{2}}$$

where *n* is the number of sample points, *v_l_* is the actual measured data, and $\hat{v_{l}}$ is the predicted value calculated from the model segmentation results. *v̄_l_* is the average of all actual measurements.

## 3 Experiments and results

### 3.1 Nerfacto-based 3D reconstruction validation experiments

To evaluate the effectiveness of the Nerfacto-based 3D reconstruction method in plant phenotypic parameter extraction, we choose to use the point clouds generated by the mainstream Colmap (Schonberger and Frahm, 2016) open-source software and the 3DF Zephyr commercial software as the reference for the geometric extraction results. Colmap, known for its open-source nature and wide range of applications, is capable of providing highly accurate reconstruction results of point cloud data, while 3DF Zephyr, an industry-leading commercial software, is widely recognized for its fast processing and high-quality reconstruction capabilities. By comparing these reference point clouds, we aim to validate the reconstruction performance and applicability of the Nerfacto (Tancik et al., 2023) method in complex plant scenes, thus providing a new solution for the efficient acquisition of point cloud data.

**Figure 7:**
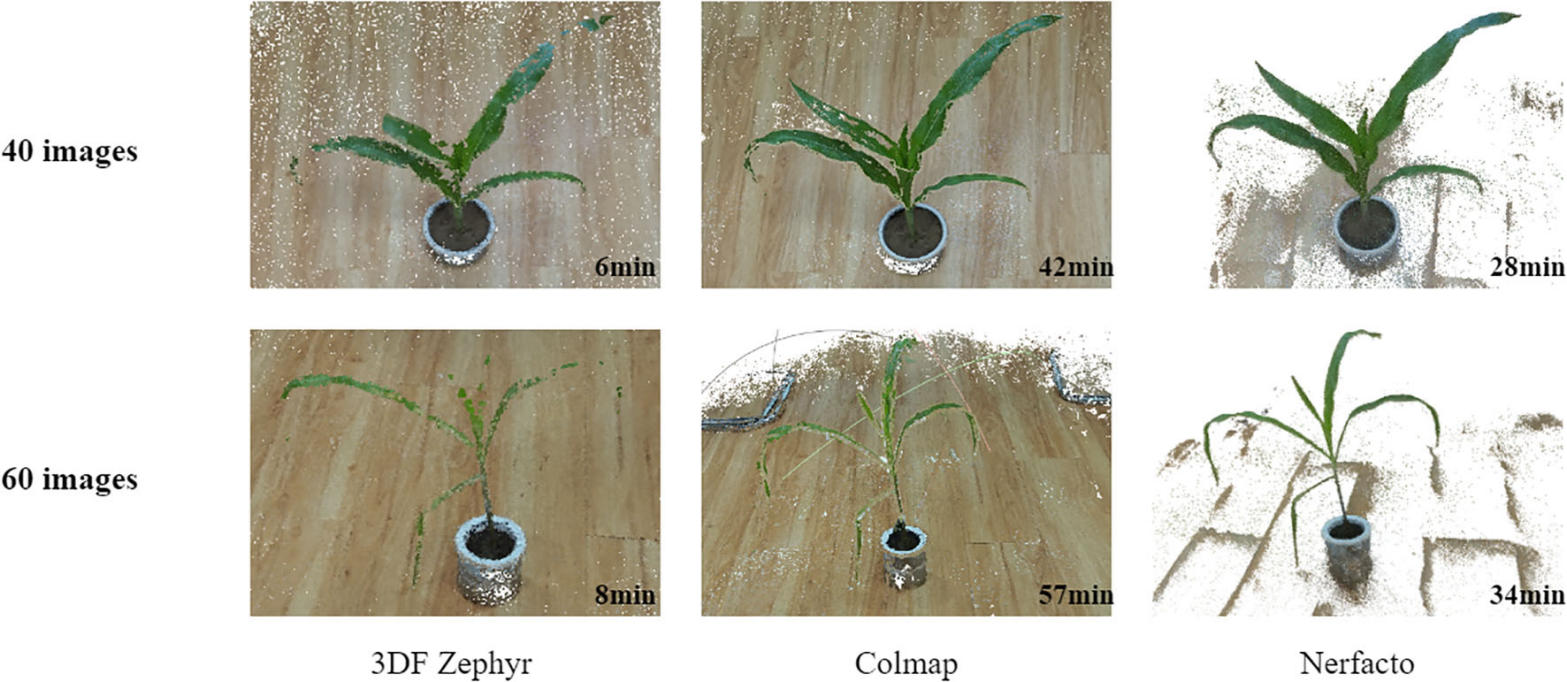
Visualization results of 3D reconstruction using Nerfacto, Colmap open source software and 3DF Zephyr commercial software at different numbers of images and the time taken for reconstruction.

In the experiments, we provide a detailed analysis from the following three aspects: reconstruction quality under limited viewpoints, visualization of 3D point cloud models, and reconstruction time. The experiments are divided into two groups: one using 40 images and the other using 60 images. As shown in **Figure 7**, Nerfacto is capable of generating high-quality 3D models even under conditions with limited viewpoints. In contrast, 3DF Zephyr and Colmap are significantly more limited in their reconstruction, and the point cloud models may be less complete and less detailed. In terms of reconstruction time, Nerfacto takes longer in the training phase and is less affected by the number of images. Colmap’s dense reconstruction is very slow and consumes a lot of time mainly in stereo matching. In contrast, 3DF Zephyr’s reconstruction is faster, but the generated dense point cloud is sparse and cannot capture all the details, and there may be voids and breaks in the point cloud.

Therefore, Nerfacto can be taken as a basic tool for 3D reconstruction of plants in agriculture. Later experimental results show that its high-quality reconstruction can help researchers analyze and characterize plant structures more precisely, providing strong support for plant phenotyping and related research.

### 3.2 Comparisons of different point cloud segmentation methods

#### 3.2.1 Ablation study

In order to fully evaluate the performance and effectiveness of our proposed point cloud segmentation method, we conducted systematic ablation experiments on the Tomato dataset, as shown in **Table 1**. Our aim is to validate the contribution of each key component of the method to the final segmentation results and provide a basis for model optimization. Our ablation experiments verify the effectiveness of individual components in point cloud segmentation by removing or replacing these modules one by one.

**Table 1:** Comparison of results from network model ablation experiments on the tomato point cloud dataset.

Ablate	mIoU (%)	Δ	Params (M)	FLOPS (G)
PointSegNet(ours)	92.525	−	1.33	4.73
− RSA	91.64	0.885	1.068	4.63
− CAM-SE	92.1	0.425	1.32	4.76
− EAFP	91.21	1.315	1.24	4.6
− ResMLP	91.96	0.565	1.152	2.72
− RSA & EAFP	89.73	2.795	0.981	4.5

− denotes removing from baseline.

The ablation study results demonstrate that the complete PointSegNet model achieves a mIoU of 92.525%, indicating its high segmentation performance. Removing individual modules leads to a decrease in model performance: removing the Relative Spatial Attention (RSA) module results in a 0.885% drop in mIoU; removing the channel attention module results in a 0.425% drop; replacing the Edge-Aware Feature Propagation (EAFP) module with the FP module from PointNet results in a 1.315% drop; and replacing the ResMLP module with the SA module from PointNet results in a 0.565% drop. Notably, removing both the RSA and EAFP modules simultaneously causes a significant performance drop of 2.795%. These results indicate that the RSA and EAFP modules make significant contributions to the model’s performance, while the channel attention and ResMLP modules also provide some performance improvement. Although removing these modules reduces the number of parameters and computational complexity to some extent, the significant performance drop demonstrates that these modules are essential for enhancing the segmentation performance of the PointSegNet model.

#### 3.2.2 Point cloud segmentation results with different datasets

To comprehensively evaluate the performance of PointSegNet, we compared it with several advanced models, including PointNet++ (Qi et al., 2017), PCT (Guo et al., 2021), PointMLP (Ma et al., 2022), SPoTr (Park et al., 2023), and PointVector (Deng et al., 2023). **Tables 2**, **3** presents the quantitative comparison results for maize organ segmentation, where PointSegNet excels in efficiency metrics such as model parameters and throughput. In terms of segmentation metrics, including Intersection over Union (IoU), F1 score, and recall, PointSegNet surpasses the second-best model by approximately 1%. Although its precision is slightly lower than that of the PointVector model, PointSegNet’s model parameter count and computational complexity are only one-third of those of the PointVector model. **Table 4** further demonstrates that PointSegNet performs exceptionally well in handling plants with complex structures, such as tomatoes and soybeans, outperforming other models across all four average quantitative metrics. Experimental results indicate that PointSegNet exhibits strong robustness and versatility, reflecting its excellent generalization ability and feature extraction accuracy, which is attributed to its optimized model structure, effective feature fusion, and superior data preprocessing techniques in this paper.

**Table 2:** Comparison of segmentation performance on maize point cloud datasets.

		Maize		
		Leaf	Stem	Mean
ins. IoU (%)	PointNet++	83.27	98.84	91.055
	PCT	65.92	96.63	81.275
	PointMLP	83.49	98.71	91.1
	SPoTr	85.9	98.93	92.415
	PointVector	86.5	99.08	92.79
	Ours	**88.26**	**99.2**	**93.73**
Precision (%)	PointNet++	94.05	99.22	96.635
	PCT	69.88	**99.54**	84.71
	PointMLP	91.82	99.4	95.61
	SPoTr	94.95	99.37	97.16
	PointVector	**96.06**	99.33	**97.695**
	Ours	94.97	99.52	97.245
F1 score (%)	PointNet++	90.97	99.42	95.195
	PCT	79.99	98.29	89.14
	PointMLP	91.25	99.35	95.3
	SPoTr	92.61	99.47	96.04
	PointVector	92.84	99.54	96.19
	Ours	93.85	**99.6**	**96.725**
Recall (%)	PointNet++	88.1	99.62	93.86
	PCT	**93.53**	97.07	95.3
	PointMLP	90.69	99.31	95
	SPoTr	90.39	99.57	94.98
	PointVector	89.83	**99.75**	94.79
	Ours	92.75	99.67	**96.21**

The best and second-best results are highlighted in bold and underlined.

**Table 3:** Comparison of segmentation efficiency using PointSegNet and five other state-of-the-art networks.

	PointNet++	PCT	PointMLP	SPoTr	PointVector	Ours
Params (M)	1.74	2.88	16.71	25.56	4.07	1.32
FLOPs (G)	4.09	2.32	132.85	151.18	14	4.73

**Table 4:** Comparison of segmentation performance on tomato and soybean point cloud datasets.

		Tomato			Soybean			
		Leaf	Stem	Mean	Leaf	Stem	Branch	Mean
ins. IoU (%)	PointNet++	94.44	82	88.22	93.84	51.4	45.44	63.560
	PCT	93.07	77.19	85.13	92.79	51.61	29.17	57.857
	PointMLP	93.64	79.08	86.36	93.75	49.38	36.13	59.753
	SPoTr	94.51	83.1	88.805	94.79	57.37	43.05	65.070
	PointVector	95.8	87	91.4	94.86	62.86	48.26	68.660
	Ours	**96.45**	**88.6**	**92.525**	**95.53**	**63.14**	**51.12**	**69.930**
Precision (%)	PointNet++	96.75	90.71	93.73	96.13	66.54	71.11	77.927
	PCT	95.34	89.62	92.48	95.07	67.99	52.75	71.937
	PointMLP	95.74	90.87	93.305	96.29	62.75	57.28	72.107
	SPoTr	96.31	92.92	94.615	96.14	75.02	66.2	79.120
	PointVector	97.52	93.55	95.535	**96.79**	**75.61**	67.58	79.993
	Ours	**97.93**	**94.53**	**96.23**	96.52	73.74	**75.28**	**81.847**
F1 score (%)	PointNet++	97.14	89.82	93.48	96.83	69.94	66.36	77.710
	PCT	96.4	86.44	91.42	96.2	68.48	47.54	70.740
	PointMLP	96.72	88.06	92.39	96.7	65.84	54.36	72.300
	SPoTr	97.2	90.89	94.045	97.32	74.58	61.31	77.737
	PointVector	97.87	93.04	95.455	97.36	77.09	65.92	80.123
	Ours	**98.2**	**93.96**	**96.08**	**97.7**	**77.3**	**68.41**	**81.137**
Recall (%)	PointNet++	97.53	88.93	93.23	97.54	73.7	62.21	77.817
	PCT	97.48	83.48	90.48	97.36	68.98	43.27	69.870
	PointMLP	97.71	85.42	91.565	97.12	69.24	51.72	72.693
	SPoTr	98.11	88.95	93.53	98.52	74.15	57.1	76.590
	PointVector	98.22	92.52	95.37	97.94	78.62	**64.34**	80.300
	Ours	**98.47**	**93.4**	**95.935**	**98.9**	**81.23**	62.69	**80.940**

The best and second best results are marked in bold and underlined.

**Figure 8:**
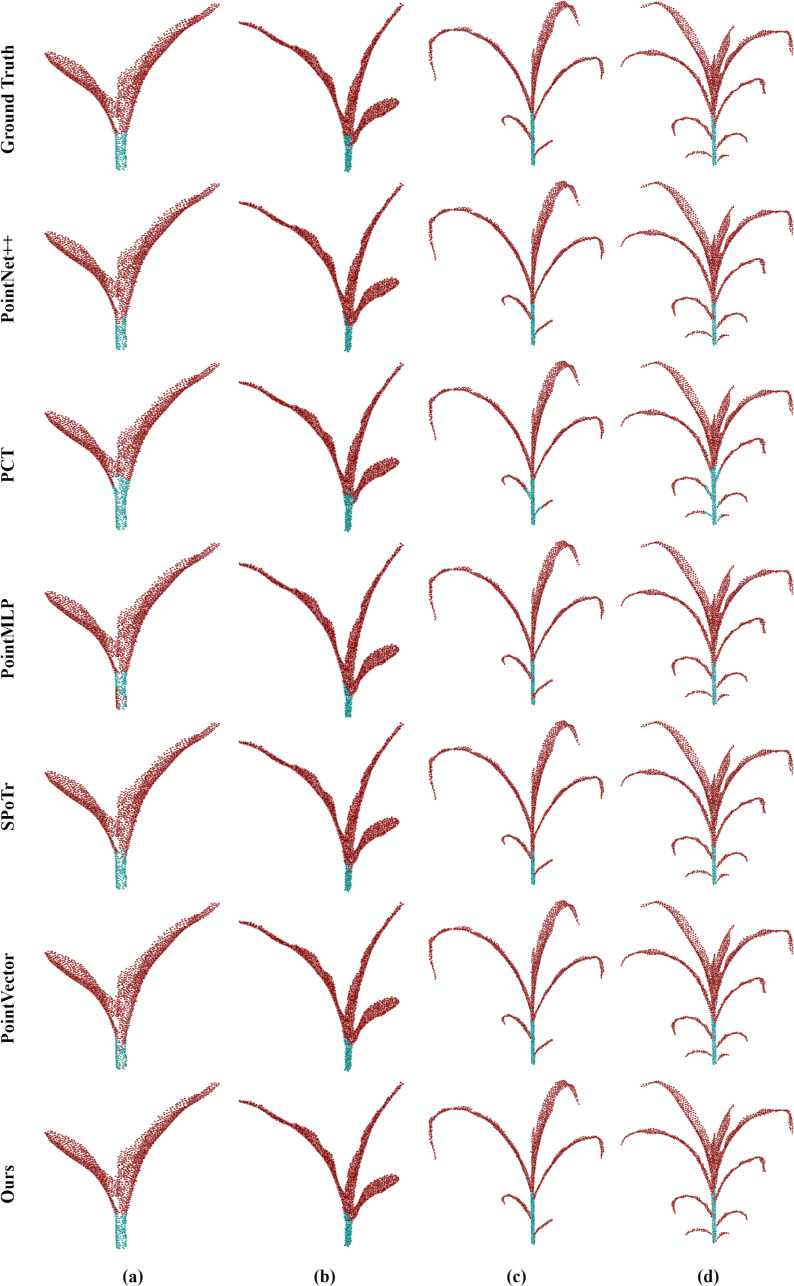
Qualitative visual analysis of organ segmentation using PointSegNet on maize point cloud datasets. **(a)**, **(b)**, **(c)**, **(d, e)** show the segmentation results and ground truth of maize point clouds for different growth cycles.

**Figure 9:**
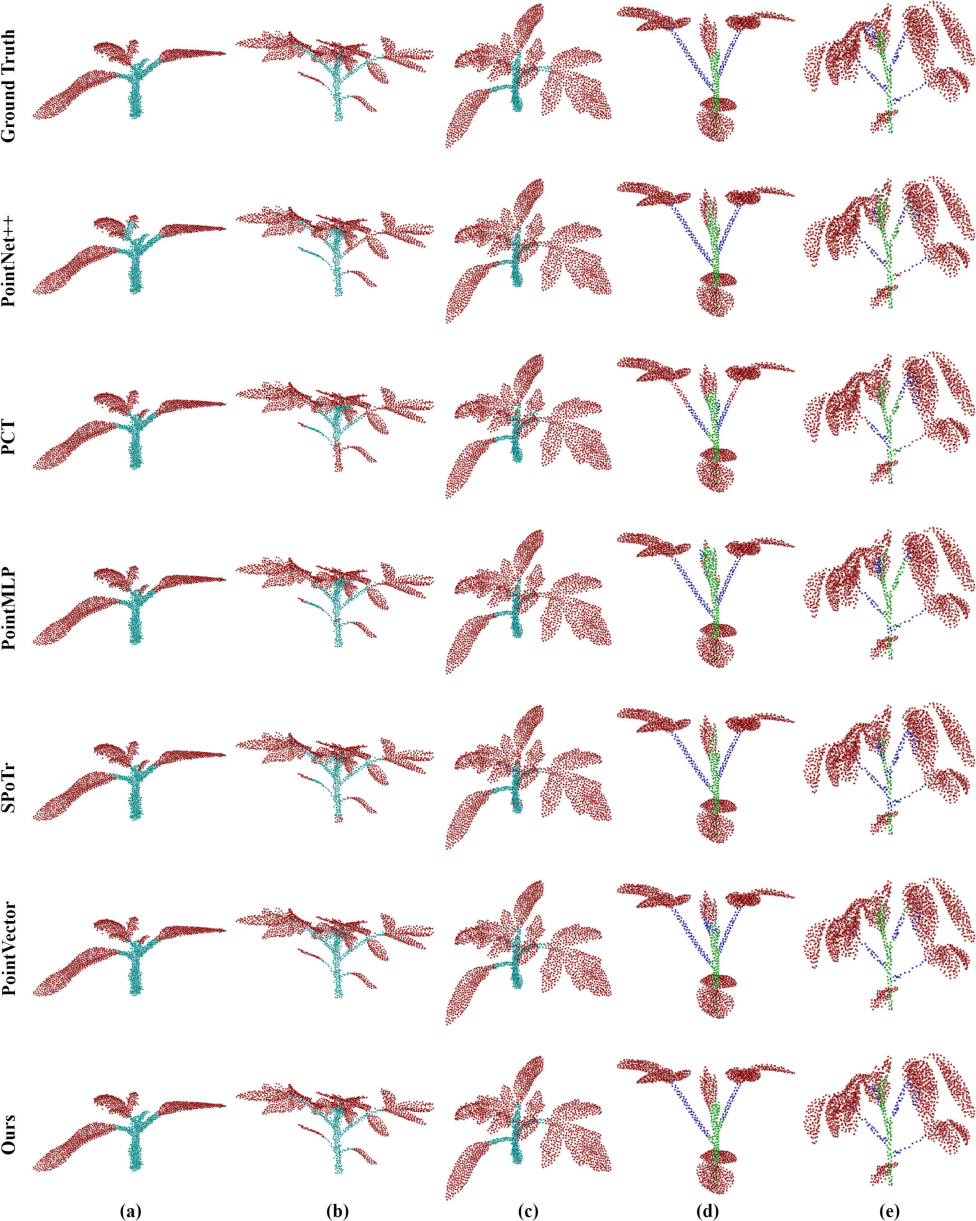
Qualitative visual analysis of complex plant organ segmentation using PointSegNet on tomato and soybean point cloud datasets. **(a–c)** show the segmentation results and ground truth for tomato, while **(d)** and **(e)** present the segmentation results and ground truth for soybean.

To show the performance of the model in various plant structures more intuitively, we carried out a detailed visualization analysis of its segmentation results. As shown in **Figure 8**, the segmentation results of maize organs are visualized and analyzed at their different growth stages. PointSegNet has high segmentation accuracy, avoiding obvious mis-segmentation or segmentation omission, and the corresponding segmented stems and leaves have clear and smooth boundaries, and accurately show the actual shapes of them, meanwhile, maintaining a high degree of consistency at different growth stages. Additionally, in **Figure 9**, we show the visualization results of point cloud organ segmentation for tomato and soybean. For tomato and soybean, due to the complexity of the plant structure, all five models except PointSegNet show mis-segmentation to varying degrees, which are mainly concentrated at the junction of stems and leaves as well as at the intersection of the main stem and branches. Especially in the dense leaf region, these models often fail to distinguish neighboring leaves due to their occlusion and overlapping correctly. In contrast, PointSegNet can effectively handle the challenges posed by these complex structures, maintains high segmentation accuracy and detail retention, and can accurately segment stems, leaves, main stems, and branches with clearer boundaries.

### 3.3 Evaluation of extracted traits based on point cloud segmentation

**Figure 10:**
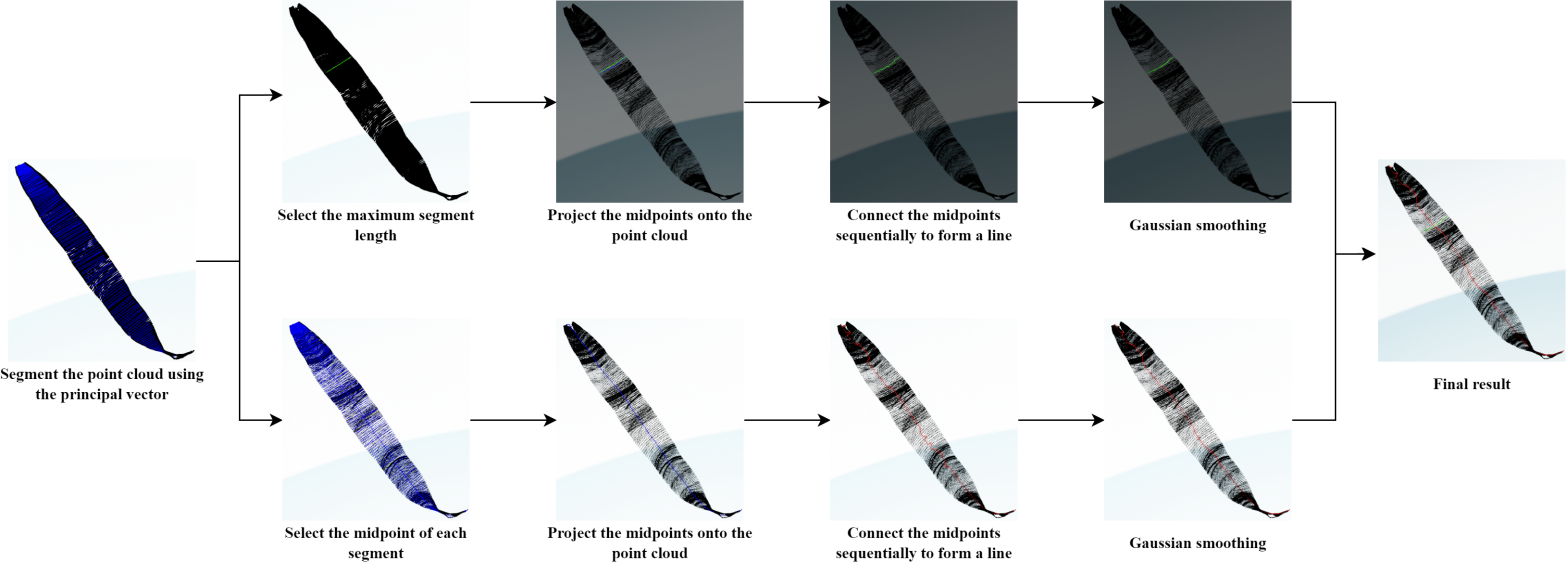
Visualization flowchart for leaf length and leaf width parameter extraction.

To validate the effectiveness of the proposed leaf length calculation method, we visualized each step of the process. As shown in **Figure 10**, we first computed the principal direction of the leaf point cloud using PCA and segmented the point cloud based on projection values. Next, we calculated the principal vector for each segment of the point cloud to identify the nearest and farthest points. Due to the curvature of maize leaves, using the distance between the nearest and farthest points directly as the leaf width is inaccurate. Therefore, we propose to generate points uniformly between these two points and projecting them onto the leaf. By connecting the nearest point, projection points, and the farthest point, the total length of the resulting segments is taken as the leaf width, which better fits the shape of the leaf. For leaf length, we project the midpoints of the nearest and farthest points in each segment onto the segment’s point cloud. We then connected the nearest points, farthest points, and projection points across the entire leaf point cloud to determine the total length of the leaf. Due to improper setting of the number of segments and the number of uniform point insertions, the resulting curves might exhibit excessive undulations, failing to accurately reflect the leaf parameters. To address this, we apply Gaussian smoothing to these points by using one-dimensional Gaussian filtering along the x, y, and z axes, respectively, to enhance data continuity and smoothness.

**Figure 11:**
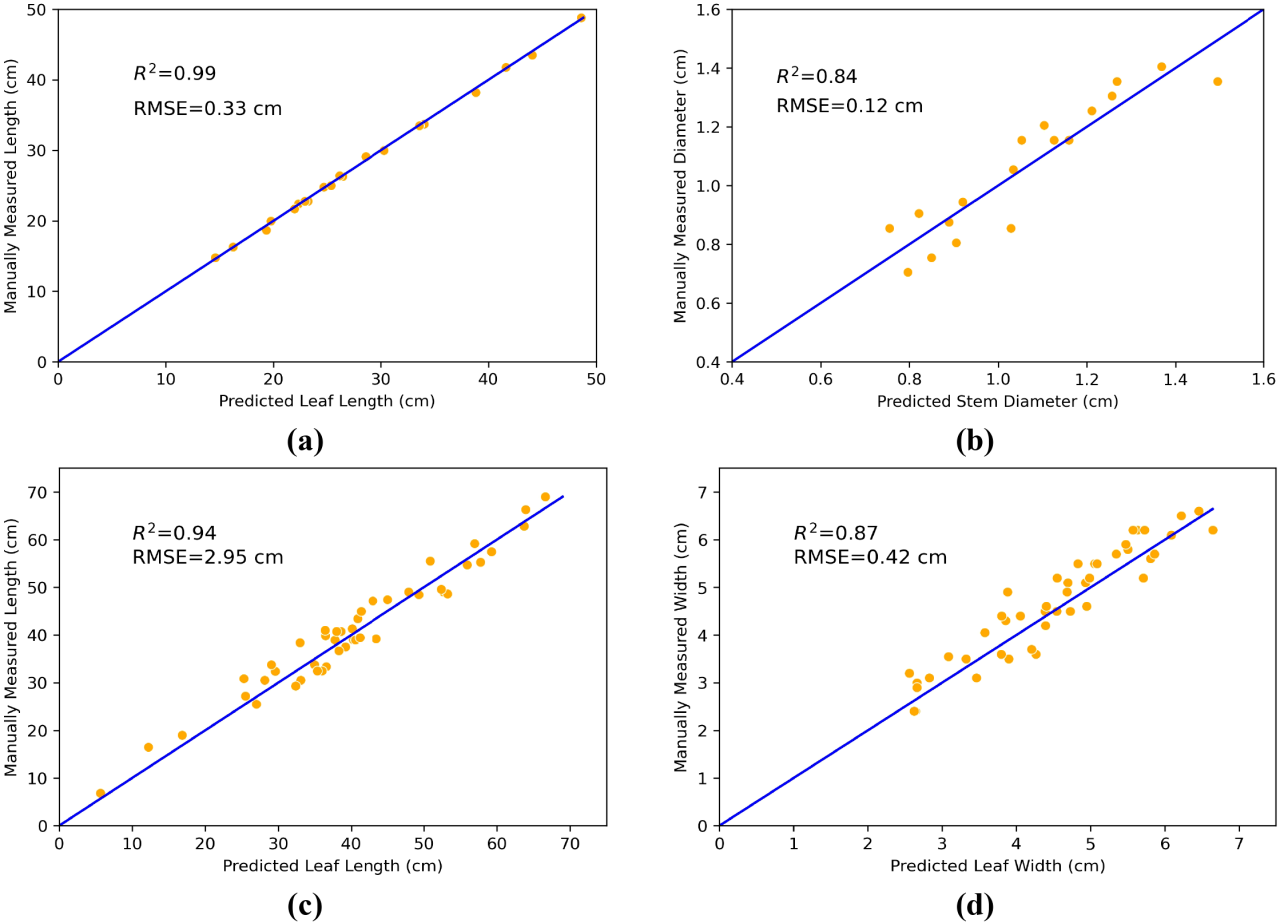
Comparison of extracted phenotypic parameters based on maize point cloud segmentation with measured values. **(a)** stem height, **(b)** stem diameter, **(c)** leaf length, and **(d)** leaf width.

To assess the performance of point cloud segmentation results in plant phenotypic parameter extraction, we compared the measurement results obtained using our proposed method with those obtained through manual measurement. As shown in **Figure 11a**, the validation results about plant stem height produced *R* ^2^ and RMSE values of 0.99 and 0.33 cm, respectively. **Figure 11b** shows that the validation of plant stem diameters resulted have *R* ^2^ and RMSE values of 0.84 and 0.12 cm, respectively. The validation results for leaf length and width are presented in **Figures 11c, d**, where the leaf length has an *R* ^2^ value of 0.94 and an RMSE value of 2.95 cm, and the leaf width has an *R* ^2^ value of 0.87 and an RMSE value of 0.42 cm. These results indicate that the proposed method in this paper has high accuracy in extracting plant phenotypic parameters, effectively capturing and reflecting the actual characteristics of the plants.

## 4 Discussion

This paper employs the Nerfacto method for the three-dimensional reconstruction of maize, extracting point clouds from implicit neural radiance fields. The Nerfacto method has low requirements for input data and is capable of generating high-quality 3D models with limited viewpoints and images, making it adaptable to various scenes and backgrounds, and suitable for the reconstruction of different plant types. During the image acquisition process, uneven lighting or strong reflections may lead to issues such as shadows, glare, or overexposure in the images, which could introduce errors in the reconstruction results and affect the final quality of the 3D model. Therefore, we will consider the impact of environmental lighting during data acquisition and processing, and employ appropriate image preprocessing techniques, such as exposure correction, to mitigate the interference of these factors on phenotyping measurements. Although the training and inference processes of Nerfacto can be completed using a single consumer-grade GPU, the entire procedure typically requires 20 to 30 minutes. Particularly when processing high-resolution images, the computational resource consumption and time required increase significantly. To address this issue, we plan to utilize CUDA programming to implement part or all of the computationally intensive tasks, thereby improving processing efficiency and accelerating the training process.

In our study, the PointSegNet model, which downsamples plant point clouds to 2048 points, has demonstrated excellent segmentation performance. However, for plants with complex structures, significant downsampling can compromise the original structural details, leading to information loss and makes it challenging for the network to learn useful features. Increasing the number of points in the plant point cloud can better preserve plant detail information, thereby improving segmentation accuracy. Nevertheless, this also introduces new challenges: higher point cloud resolution results in a substantial increase in the network’s parameter count and computational load, and requires significant computational resources and time for training and inference. This may potentially lead to issues with insufficient computational resources and reduced efficiency in practical applications. Therefore, finding the optimal balance between model performance and computational resource requirements is a critical direction for future research. The effectiveness of plant point cloud segmentation generally will be improved with the richness of the dataset. However, point-by-point annotation is a time-consuming and labor-intensive task, which limits the acquisition of large-scale, high-quality datasets. To enhance model performance with limited data, we will explore techniques such as semi-supervised learning, self-supervised learning, and transfer learning in future work. These approaches will not only accelerate model development and application but also reduce data annotation costs and time investment, thus providing substantial support for the advancement of agricultural phenotype measurement technologies.

In the process of extracting plant phenotype parameters, such as stem height, stem diameter, leaf length, and leaf width, the calculation often relies on methods that are dependent on key points. Traditional methods, which rely solely on the distance between the nearest and farthest points, may not accurately fit the curvature of the leaf when it is bent, resulting in an underestimation of leaf length. Our improved approach addresses this issue by segmenting the leaf along its principal direction, thereby enhancing the accuracy of the leaf curve fitting and resulting in more precise measurements of leaf length and width. However, excessive segmentation may lead to an overly curved fit, affecting the final measurement results. Therefore, it is essential to set the number of segments appropriately to ensure that the fitted curve closely approximates the actual shape of the leaf while maintaining measurement accuracy. Additionally, the presence of outliers is a common and significant issue. Outliers can arise from various sources, such as errors in point cloud acquisition equipment, environmental interference, similar situations occur in color between the plant and background, or errors in dataset annotations. These outliers may introduce substantial errors in phenotype parameter calculations, thereby affecting the final measurement results. Future research should focus on methods to prevent outlier interference during phenotype parameter computation to enhance the accuracy and reliability of measurements.

## 5 Conclusion

In this paper, a plant stem and leaf segmentation and phenotypic parameter extraction is proposed. Firstly, a novel 3D reconstruction method, Nerfacto, is employed to plant 3D reconstruction to explore the extraction of point clouds from implicit neural radiance fields. This approach provides a new avenue for obtaining point cloud data and reduces the reconstruction difficulty associated with complex plant scenes by using traditional methods. Secondly, a lightweight plant organ point cloud segmentation network, PointSegNet, is developed. This network features an efficient encoder-decoder structure that significantly enhances segmentation accuracy while maintaining low parameter counts and computational complexity. Experimental results on maize, tomato, and soybean datasets demonstrate that PointSegNet outperforms existing advanced networks in segmentation performance, with higher accuracy and robustness. Lastly, the PCA-based methods are proposed for calculating leaf length and leaf width parameters by determining the primary direction of the leaf point cloud. Experimental results indicate that the proposed methods achieve high precision in extracting phenotypic parameters such as stem length, stem diameter, leaf length, and leaf width. In the future, we aim to provide stronger support for plant phenotyping research and agricultural applications by continuously optimizing and expanding these technologies to develop automated tools in smart agriculture.

## Data Availability

The raw data supporting the conclusions of this article will be made available by the authors, without undue reservation.
